# Evolutionary significance of metabolic network properties

**DOI:** 10.1098/rsif.2011.0652

**Published:** 2011-11-30

**Authors:** Georg Basler, Sergio Grimbs, Oliver Ebenhöh, Joachim Selbig, Zoran Nikoloski

**Affiliations:** 1 Institute of Biochemistry and Biology, University of Potsdam, Karl-Liebknecht-Str. 24-25, 14476 Potsdam, Germany; 2 Institute of Complex Systems and Mathematical Biology, Department of Physics, University of Aberdeen, SUPA, Aberdeen AB24 3UE, UK; 3 Max Planck Institute for Molecular Plant Physiology, Am Mühlenberg 1, 14476 Potsdam, Germany

**Keywords:** metabolic networks, significance, randomization, null model, centrality

## Abstract

Complex networks have been successfully employed to represent different levels of biological systems, ranging from gene regulation to protein–protein interactions and metabolism. Network-based research has mainly focused on identifying unifying structural properties, such as small average path length, large clustering coefficient, heavy-tail degree distribution and hierarchical organization, viewed as requirements for efficient and robust system architectures. However, for biological networks, it is unclear to what extent these properties reflect the evolutionary history of the represented systems. Here, we show that the salient structural properties of six metabolic networks from all kingdoms of life may be inherently related to the evolution and functional organization of metabolism by employing network randomization under mass balance constraints. Contrary to the results from the common Markov-chain switching algorithm, our findings suggest the evolutionary importance of the small-world hypothesis as a fundamental design principle of complex networks. The approach may help us to determine the biologically meaningful properties that result from evolutionary pressure imposed on metabolism, such as the global impact of local reaction knockouts. Moreover, the approach can be applied to test to what extent novel structural properties can be used to draw biologically meaningful hypothesis or predictions from structure alone.

## 1. Introduction

The central findings in network-based research suggest that there exist simple mechanisms directing the evolution of both engineered and natural networks [1–10]. However, the relation between the functions of a biological system and its network properties is hardly understood. Therefore, the advantage of using network representations for positing meaningful hypotheses about biological systems remains largely debatable [11].

Properties of biological systems arise from two fundamental origins: *physical principles*, universally constraining the feasibility of biochemical processes, and *evolutionary pressure*, bearing the specific functional abilities required for an organism's vitality [12]. The former comprise well-understood physical laws, such as mass balance and thermodynamics, which constitute the basic requirements imposed on all living systems. In contrast, evolution depends on the interplay of complex phenomena, such as adaptation to environmental changes, symbiosis and biodiversity of populations [13,14], leading to diverse cellular functions. Consequently, the unique properties related to the functions of a biological system are a result of its evolutionary history.

Explaining cellular behaviour through network representations and their properties is a key challenge of modern biology. While many structural properties of metabolic networks are similar to those of other complex networks [10], it is unclear whether they are a consequence of the evolutionary history or merely arise as a result of general physical principles. Here, we apply a randomization method to determine which properties of metabolic networks, represented as bipartite metabolite-reaction graphs, may result from evolutionary pressure. This is an essential step in understanding the relation between the functional characteristics of biological systems and their network representations.

The common approach for estimating the relevance of a network property is to determine the statistical significance (*p*-value) by comparing the value of the property in the investigated network with those in the null-model distribution obtained from randomized networks [15]. Clearly, the significance of a property strongly depends on the chosen null model, which should be constrained to preserve universal network properties [16,17]. Since the *p*-value is the probability that the value of a property originates from the null-model distribution, a statistically significant property is likely to have emerged from some non-arbitrary process influencing network evolution independently of the imposed constraints.

**Figure 1.**
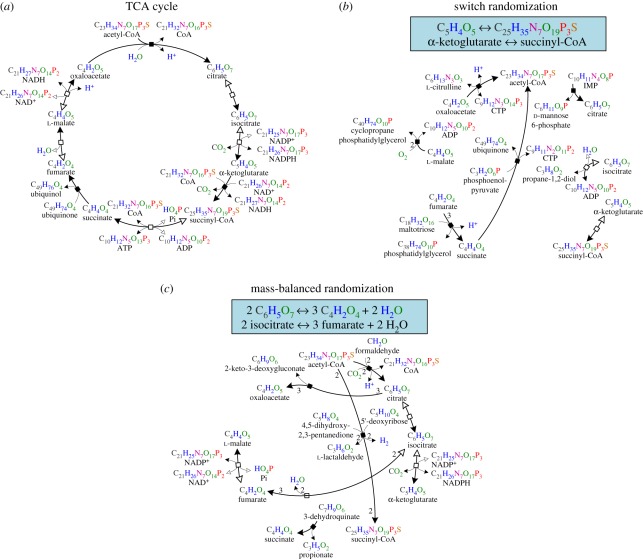
Illustration of how switch and mass-balanced randomization of the genome-scale metabolic network of *Escherichia coli* affect the TCA cycle. (*a*) The TCA cycle in *Escherichia coli*, consisting of eight reactions and 22 compounds. Compound names are shown with corresponding sum formulas, irreversible reactions are represented by solid squares and reversible reactions are denoted by blank squares. Internally, a reversible reaction is represented by one vertex for each direction, in order to adequately model the substrate–product relationships (see §4). (*b*) Reactions involving metabolites from the TCA cycle (bold arrows and names) after applying switch randomization. The degrees of compounds and reactions are preserved, but the generated reactions violate fundamental physical constraints (see inlay). Note that the reactions shown are obtained from randomization of the entire network of *Escherichia coli*; the degrees therefore do not correspond to those shown in (*a*). (*c*) All reactions obtained by mass-balanced randomization are chemically feasible owing to balanced atom masses and realistic thermodynamic energy ranges, as indicated by the sum formulas and stoichiometric coefficients (thermodynamic data not shown).

In virtually all network-based studies [18–24], a Markov-chain switching algorithm, *switch randomization*, has been employed to determine the significance of network properties by generating randomized networks with preserved degree sequence. Its motivation stems from the finding that heavy-tail degree distributions are a universal feature of complex networks. This generic null model can be applied to any type of network, and guarantees the independence of an identified property from vertex degrees. We demonstrate how switch randomization affects the citric acid (TCA) cycle, a central respiratory metabolic pathway of outstanding importance for aerobic organisms (figure 1*a*): two reactions substrate_1_ → product_1_ and substrate_2_ → product_2_ are substituted with new reactions substrate_1_ → product_2_ and substrate_2_ → product_1_, ensuring that the vertex degrees remain unchanged (figure 1*b*). Since chemical feasibility is disregarded, a reaction that converts *α-ketoglutarate* into *succinyl-CoA* may be generated, where several atoms are created out of nothing. Hence, it remains hypothetical to what extent the properties, identified as significant with this method, relate to the function of the network, as they could well result from universal physical constraints imposed during network evolution.

## 2. Results

### 2.1. Measuring evolutionary significance

To identify the properties that originate from evolutionary pressure, a network should be compared with random networks that evolved free of evolutionary pressure, but persistently satisfy all relevant physical constraints. As this is practically impossible to simulate, we apply our recent method for randomizing metabolic networks while preserving mass balance of the biochemical reactions [25]. A reaction *r* with substrate set *S* and product set *P* is mass balanced if the number of substrate atoms equals the number of product atoms:2.1
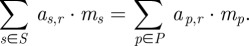
where *m*_*s*_, *m*_*p*_ are the vectors of sum formulas of *s* and *p*, respectively, and *a*_*s*,*r*_, *a*_*p*,*r*_ their stoichiometric coefficients (see §4). The mass-balanced randomization of the TCA cycle does not violate this basic physical constraint, as shown in figure 1*c*.

**Figure 2.**
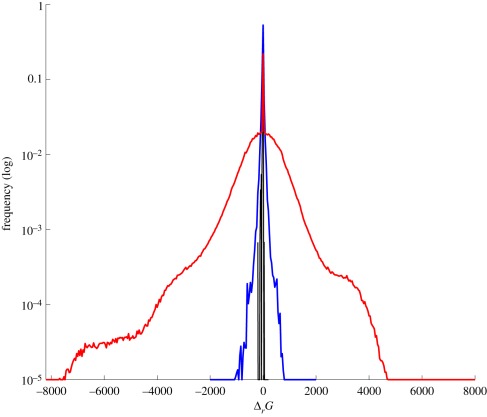
Distributions of Gibbs-free energy changes under standard conditions (*Δ*_*r*_*G*^0^ ) in *Escherichia coli* (black), and averaged over 10^4^ mass-balanced (blue) and switch randomized (red) networks. Energy changes in *E. coli* have a mean of 7.5 and standard deviation 15.1; mass-balanced randomized networks have a similar mean of 6.5 and standard deviation 53.5. In contrast, switch randomization generates implausible energy ranges with a mean of 32.5 and standard deviation 847.3.

Thermodynamic properties, reflecting the energy change of reactions, constitute another important physical requirement for metabolic networks. As shown in figure 2, the reactions generated by mass-balanced randomization of the *Escherichia coli* network are characterized by plausible Gibbs-free energy changes under standard conditions (pH = 7, *T* = 298.15 K, see the electronic supplementary material) [26]. In contrast, switch randomization results in unrealistic energy ranges. By preserving mass balance and thermodynamic properties during randomization, our null model imposes realistic physical constraints on the generated randomized networks. This ensures that the significant properties are independent of the fundamental physical requirements, and instead are likely to result from evolutionary pressure. Therefore, we refer to the statistically significant properties under the proposed null model as *evolutionary significant*.

**Figure 3.**
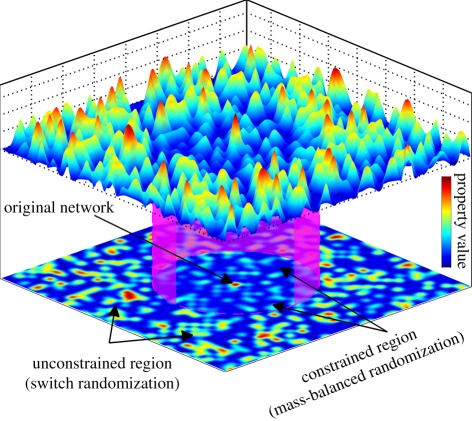
Illustration of a landscape of property values over all randomized networks. The property of the original network stands out from the inner region of constrained networks, but becomes inconspicuous in the outer region of unconstrained networks. Therefore, only by comparison with the constrained networks, one may detect the evolutionary significance of the property.

For illustration, consider a landscape formed by the values of any given property over all randomized networks (figure 3). The constrained networks, obtained by mass-balanced randomization, carve out a region in the vicinity of the original network that is embedded in the region of unconstrained networks resulting from switch randomization. As these regions exhibit different distributions of values, illustrated by different magnitudes of the peaks, an evolutionary significant property may only be identified when comparing the property of the original network with the constrained region.

### 2.2. Biosynthetic capabilities

To verify our approach, first we determine the evolutionary significance of the scope size distribution in the genome-scale metabolic networks of six model organisms: *Bacillus subtilis*, *E. coli* (bacteria), *Saccharomyces cerevisiae* (fungi), *Chlamydomonas reinhardtii* (protista), *Arabidopsis thaliana* (plantae) and *Homo sapiens* (animalia; see §4). The scope represents the set of compounds that can be produced in a metabolic network from a given set of initial nutrients [27]. We determine the scope size distribution of each network by repeatedly calculating the scope for 5000 randomly chosen sets of nutrient compounds, one set at a time, according to the following procedure: (i) from the initial set of nutrients, determine the reactions for which all substrates are contained in the nutrient set; (ii) add the products of these reactions; and (iii) repeat the procedure, until no more products can be added (see electronic supplementary material, §1.3).

The scope size distribution characterizes the biosynthetic capability of a network and has been shown to exhibit a strong correlation with the evolutionary history of organisms [28,29]. After applying mass-balanced randomization to the six networks, we compare the scope size distributions of each organism and its randomized network ensemble, and determine *p*-values using the Kolmogorov–Smirnov test (see §4). We find the scope size distributions to be evolutionary significant for all studied organisms (*p*-values <10^−49^; electronic supplementary material, table S4 and figure S1), which demonstrates that our method correctly identifies the interdependence of the network property and its evolutionary background.

### 2.3. Small-world property

In the following, we focus on determining the evolutionary significance of salient network properties that have been extensively studied in complex network research and are prominently applied in biological studies. In particular, we analyse the small-world property [30], defined by a large clustering coefficient in conjunction with small average path length, and the metabolite degree distribution [8] (electronic supplementary material, table S2). We find that the clustering coefficient is significant in all species (*p*-values < 10^−5^), regardless of the applied null model. On the other hand, the average path length is evolutionary significant with *p*-values < 0.025 in all species (electronic supplementary material, table S4). With switch randomization, this property is significant (*p*-values < 10^−5^) in all but *S. cerevisiae* (*p*-value =0.77; electronic supplementary material, table S5).

**Figure 4.**
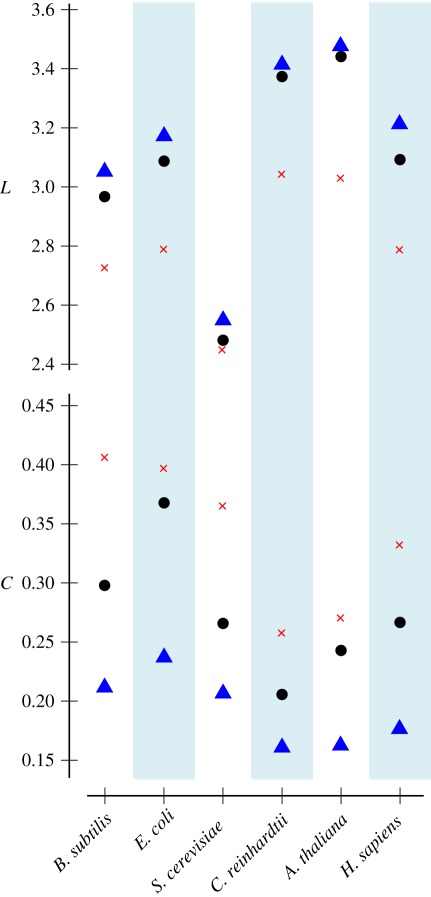
Characteristic path lengths (*L*) and clustering coefficients (*C*) of the six investigated metabolic networks (black dots) and averaged values of their mass-balanced (blue triangles) and switch randomized (red crosses) ensembles. Compared with the mass-balanced null model, characteristic path lengths are small and clustering coefficients large in all six organisms, confirming the small world hypothesis. Contrarily, in comparison to the switch based null model, characteristic path lengths are large and clustering coefficients small. The standard deviation is below 0.02 for each randomized distribution.

More importantly, we may now assess the importance of the small-world phenomenon by determining whether this property is more pronounced in the analysed networks when compared with their randomized variants. Interestingly, in each species we find that the average path length is smaller and the clustering coefficient is greater than the values of the respective properties obtained from mass-balanced randomization (figure 4). This finding indicates that the small-world property is independent of physical constraints, and thus likely to be of evolutionary importance for metabolic networks. By contrast, when comparing the networks with their switch randomized ensembles, we arrive at a contrary conclusion—larger average path lengths and smaller clustering coefficients are prominent in real-world metabolic networks. Therefore, the results from switch randomization suggest that metabolic networks are the opposite of small worlds, disproving the small-world hypothesis. Moreover, this finding hints at two major hazards of network null models: (i) the results obtained crucially depend on the model chosen, and (ii) the application of a generic null model that provides an unrealistically constrained environment may lead to counterintuitive results.

### 2.4. Degree distributions

Next, we analyse the metabolite degree distributions, where the degree of a metabolite is the number of reactions it is involved in either as a substrate or a product. The degree can be interpreted as metabolite specificity, with highly specific metabolites occurring in only few reactions. To our knowledge, the significance of degree distributions was never studied, since switch randomization is unsuited for this task. The degree distributions of all six organisms are evolutionary significant (*p*-values < 10^−17^; electronic supplementary material, table S4 and figure S3), suggesting that the patterns of metabolite specificities across different organisms emerge as a consequence of their evolutionary history, and not from the imposed physical constraints. This finding complements the well-known evolutionary requirement of a network architecture that is robust to random errors, as exhibited by the heavy-tail degree distributions [8].

### 2.5. Reaction centrality

Finally, we propose a measure for determining the global importance of individual reactions, which is based on a centrality index previously used in sociological studies [31] (also referred to as Hubbell Index). For two reactions *r*_*i*_ and *r*_*j*_ , we define the dependence of *r*_*j*_ on *r*_*i*_ as the largest ratio by which *r*_*i*_ contributes to the overall production of an intermediary *c* (i.e. a compound that is produced by *r*_*i*_ and consumed by *r*_*j*_ ): *ω* (*r*_*i*_, *r*_*j*_ ) = max_*c*_*d*_in_(*c*)^−1^, where *d*_in_(*c*) is the in-degree of *c*, which is the total number of reactions producing *c*. Note that this definition corresponds to the strength of impact of a knockout of *r*_*i*_ on *r*_*j*_ , where *ω* (*r*_*i*_, *r*_*j*_ ) = 1, if *r*_*j*_ becomes inoperable upon knockout of *r*_*i*_ (e.g. if *r*_*i*_ and *r*_*j*_ are neighbours in a linear chain of reactions), and *ω*(*r*_*i*_, *r*_*j*_ ) ∼ 0, if the intermediaries required by *r*_*j*_ can be produced by many other reactions in the network.

The global impact of the knockout of a reaction on the entire network, which we call *reaction centrality*, is2.2
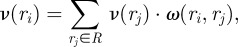
where *R* is the set of all reactions in the network, and *ω*(*r*_*i*_, *r*_*j*_) = 0, if *r*_*i*_ and *r*_*j*_ do not share any intermediary compound (i.e. *r*_*i*_ and *r*_*j*_ are not directly connected). This measure accounts for the direct dependencies between reactions through their intermediary compounds, as well as the global importance of the affected reactions: a knockout may affect only few other reactions directly, but can still have a large impact on the network, if an important reaction is affected indirectly (e.g. the knockout of a reaction at the beginning of a linear chain that leads to a reaction producing many important compounds).

Equation (2.2) can be written in matrix form as *Aν* = *ν*, where *A*_*i*,*j*_ = *ω*(*r*_*i*_, *r*_*j*_). In order to solve this eigenvalue problem, we need to ensure that the inverse of *A* exists, which can be achieved by the PageRank transformation [32]. In particular, the transformed matrix *A*′ is obtained by normalizing the columns of *A* and applying a damping factor *d*:
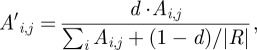
which yields the Markov chain represented by *A*′ ergodic, as the corresponding network is strongly connected, and ensures that the largest eigenvalue is 1. In order to minimize the diluting effect of the damping factor on the topology of *A*, we choose *d* = 0.99. The eigenvector *ν* corresponding to the eigenvalue 1 of *A*′ then contains the global centrality values of the reactions in the network, where *ν*(*i*) corresponds to the reaction centrality of the *i*th reaction. The calculation for large networks is tractable using a Fortran implementation of the Implicitly Restarted Arnoldi Method [33].

We determine a *p*-value for each reaction by comparing its centrality value in the original network with those obtained from mass-balanced randomized networks while preserving the reaction itself. In order to estimate the effect of evolutionary pressure towards high centrality values, we apply a one-sided test with the null hypothesis that the values obtained from randomization are at least as large as the values of the original reactions (see §4).

Table 1 shows the reactions that have a significant centrality (*p*-value ≤ 0.025) in at least three of the analysed species (see the electronic supplementary material, table S7 for a complete list). The references provide evidence that each reaction is of outstanding importance for metabolism, as demonstrated by their evolutionary ubiquity, severity of knockout or inhibition effects, and clinical applications. For instance, catalase (EC 1.11.1.6) inactivation was shown to have severe effects on the lifespan of *S. cerevisiae* cells [34]. Superoxide dismutase (EC 1.15.1.1) is essential for defense against oxygen toxicity and aerobic growth in eukaryotes [36,37], and is involved in a multitude of diseases [38]. Carbonic anhydrase (EC 4.2.1.1) fulfil diverse metabolic functions in organelles, tissues and membranes of virtually all species, is used as a drug target for various diseases and is one of the evolutionary oldest enzymes [39–45]. The numerous experimental corroborations suggest that the proposed centrality index, in conjunction with the evolutionary significance determined by using our null model, could be used to predict enzymes responsible for maintaining organismal viability solely from the network structure.

**Table 1.** Enzymes catalysing the reactions with highly significant centrality across species. All reactions with centrality *p*-values <0.025 in at least three of the following species: *Bacillus subtilis* (BS), *Escherichia coli* (EC), *Saccharomyces cerevisiae* (SC), *Chlamydomonas reinhardtii* (CR), *Arabidopsis thaliana* (AT) and *Homo sapiens* (HS). A checkmark indicates that the reaction catalysed by the enzyme has a significant centrality in the corresponding species; a hyphen indicates not significant; n.a. indicates the corresponding enzyme is not annotated for the species.

enzyme	EC no.	BS	EC	SC	CR	AT	HS	references
catalase	1.11.1.6	✓	✓	✓	—	✓	n.a.	[34,35]
superoxide dismutase	1.15.1.1	✓	✓	n.a.	—	✓	✓	[36–38]
carbonic anhydrase	4.2.1.1	✓	—	✓	✓	✓	n.a.	[39–45]
l-arabinose isomerase	5.3.1.4	✓	✓	n.a.	n.a.	n.a.	✓	[46,47]
phosphoglycerate mutase	5.4.2.1	✓	—	✓	—	—	✓	[48–51]

For comparison, when repeating the analysis using switch randomization, the picture is less clear. In *S. cerevisiae*, *A. thaliana* and *H. sapiens*, 89, 27 and 14 per cent of the reactions have a *p*-value of 0.0099, rendering the analysis useless at least for the first two species. Five reactions have a significant centrality in at least two of the remaining three analysed species (electronic supplementary material, tables S6 and S8). We omit a detailed statistical analysis of these initial results, which will be necessary to draw further conclusions.

## 3. Discussion

To conclude, we proposed a novel method to reveal the relation between network properties and their evolutionary background by preserving the universal physical principles that constrain the design of metabolic networks. Any property that originates from evolutionary pressure, and thus relates to an important biological function, should not be observed in artificial metabolic networks, which evolved free of evolutionary pressure, but satisfy all relevant physical constraints. This should even hold for properties evolved from complex time-dependent phenomena, if they are reflected in the ultimately observed network.

We recognize that the proposed method only preserves mass balance and thermodynamic constraints, while other physical principles, such as electric charges, may also be relevant for metabolic network properties. Nevertheless, the considered physical constraints are the most fundamental and ubiquitous ones. Therefore, we believe that the method is a reasonable first approach to extract the biological importance of metabolic network properties. Accounting for additional physical constraints is complicated by the lack of reliable data for genome-scale metabolic networks; however, we expect such extensions to become possible in the future, which should further improve the biological relevance of the significance measure and the accuracy of the resulting predictions.

In contrast to the commonly applied switch randomization, our approach provides a realistic network background, and attributes an important evolutionary role to the small-world property and heavy-tail degree distributions. Our findings shed new light on the conclusions of previous studies, and suggest that the salient network properties are indeed a product of evolutionary pressure. Therefore, these properties carry important biological information, and can be justifiably used to generate meaningful hypotheses for experimental research.

We demonstrate that the proposed centrality index is one such network property that determines reactions important for viability of organisms. The method could therefore be used to identify candidate reactions for metabolic engineering and drug development. The results provide an impetus for addressing the long-standing doubts concerning the biological relevance of network properties. In addition, the proposed null model could be employed to verify the evolutionary assumptions in constraint-based approaches [52] and to provide an interface to synthetic biology studies.

Finally, we envision that, similar to the proposed approach for metabolic networks, specifically designed null models will be developed for other physically constrained systems, represented by gene-regulatory, protein–protein interaction and signalling networks. For instance, transcription factors depend on cis-elements and DNA-binding domains, which constrain the sequence of genes by which they are encoded. Likewise, protein interactions and signalling interactions depend on functional domains and binding sites. Development of null models which integrate the governing physical constraints of such systems will likely stimulate novel insights into the structure–function relationship in complex biological networks.

## 4. Methods

### 4.1. Genome-scale metabolic networks

We conduct our analyses on the most widely used genome-scale metabolic networks of six model organisms from all kingdoms of life: *B. subtilis* [53], *E. coli* [26], *S. cerevisiae* [54], *C. reinhardtii* [55], *A. thaliana* [56] and *H. sapiens* [57]. The sizes of the networks vary according to the complexity of the represented organisms, ranging from 855 reactions and 766 compounds (*B. subtilis*) to 2819 reactions and 2691 compounds (*H. sapiens*). Resulting from the bipartite graph reconstruction, detailed in §4.2, the number of vertices and edges varies accordingly, from 1877 vertices and 5368 edges (*B. subtilis*) to 7059 vertices and 19651 edges (*H. sapiens*). The networks further differ in their quality regarding mass balance of reactions, availability of information on reversible reactions, and the number of (strongly) connected components (electronic supplementary material, table S3): only the network of *E. coli* is fully balanced and consists of one connected component.

### 4.2. Mass-balanced randomization

To estimate the evolutionary significance of network properties, we generated 10^4^ mass-balanced randomized networks for each of the six analysed genome-scale metabolic networks. A metabolic network is represented as a directed bipartite graph *G* = (*V*_c_ ∪ *V*_r_, *E*), where *V*_c_ is the set of compound vertices, *V*_r_ the set of reaction vertices and 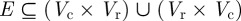 is the set of *directed* edges denoting substrate–reaction and product–reaction relationships. For a compound *c* ∈ *V*_c_, we denote by *m*_*c*_ ∈ ℕ^*n*^ its *mass vector*, i.e. the vector representation of *c* over *n* chemical elements. Here, we consider only the six most abundant elements in biological systems [58]: carbon (C), hydrogen (H), nitrogen (N), oxygen (O), phosphorus (P) and sulphur (S). The mass vector of water is then (0,^2^,0,^1^,0,0) · (C,H,N,O,P,S)^T^. Reversible reactions are represented by one reaction vertex for each direction: *r*^+^ and *r*^−^, such that *r*_in_^+^ = *r*_out_^−^ and *r*_out_^+^ = *r*_in_^−^.

In order to uniformly randomize a network while preserving mass balance, each possible mass-balanced network has to be generated with equal probability. This requires enumeration of all possible sets of substrates and products, for which equation (2.1) is satisfied. As this problem is a special case of the Knapsack problem [59], the number of possible mass-balanced networks is at least exponential in the number of compounds.

We approach the complexity of the general problem by applying a new method for mass-balanced randomization, introduced in Basler *et al.* [25]. The set of possible solutions to equation (2.1) is restricted twofold: (i) the in- and out-degrees of reactions are preserved and (ii) the substitution of compounds is limited to certain subsets, as detailed later, which allows to easily find a solution for equation (2.1). The first restriction is in line with the observation that reaction degrees are biochemically constrained by the number of interacting compounds. The second allows to divide the randomization procedure into a precalculation step and an actual randomization. As a result, the generation of a large set of mass-balanced randomized networks becomes computationally feasible.

The randomization procedure consists of two steps: In the first step, for a given metabolic network *G*, we determine the classes of mass equivalent compounds and pairs of compounds from *V*_c_(*G*). Two compounds are called mass equivalent, if the mass vector of one compound is a multiple of the other (e.g. CO_2_ and C_2_O_4_). Two pairs of compounds are called mass equivalent, if the sum of mass vectors of one pair is a multiple of the sum of mass vectors of the other pair (e.g. (CH_2_O, CO_2_ ) and (C_4_H_2_O_4_, H_2_O_2_ )). This definition ensures that the mass vectors of two compounds (and the sums of the mass vectors of two pairs of compounds) from the same mass equivalence class differ only by rational factors (e.g. 2CH_2_O + 2CO_2_ = C_4_H_2_O_4_ + H_2_O_2_ ). The precalculation of mass equivalent compounds is to be executed only once for all subsequent randomizations of the same network and renders the generation of large sets of mass-balanced randomized networks computationally feasible (see supplementary table S1 in [25] for a performance comparison to switch randomization).

In the second step, the reactions of *G* are randomized while preserving mass balance. To randomize a reaction chosen uniformly at random from *V*_r_ (*G*), its substrates and products are replaced by randomly chosen substitutes from their corresponding mass equivalence classes. When substituting an individual substrate or product, the stoichiometric coefficients of the new reaction are obtained by multiplying the corresponding previous coefficients with the earlier mentioned factor, such that equation (2.1) is satisfied. For the substitution of a pair of substrates or products, the stoichiometric coefficients satisfying equation (2.1) are determined by solving a system of *n* linear equations with two unknowns (electronic supplementary material, table S1 for examples). In case there is no solution, the substitution is not carried out. The output from this step is an (almost) uniformly randomized network in which stoichiometric coefficients are changed, edges are replaced and, consequently, the degrees of the compounds are altered [25]. The approach is in line with the observation that some fundamental properties should be fixed while carrying out the randomization—here, these are the degrees of the reaction vertices and mass balance.

### 4.3. Calculation of *p*-values

The analysed properties are calculated in the original metabolic network and in each of the 10^4^ randomized networks. For the average path length and clustering coefficient, we derive a *z*-score, 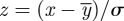, from the original value *x*, the average randomized value 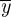, and the standard deviation of randomized values *σ*. The two-sided *p*-value is determined as *p* = 2∫_|*z*|_^∞^ 𝒩(0,^1^).

For comparing the metabolite degree and scope size distributions of the metabolic networks with their randomized versions, we apply the two-sample Kolmogorov–Smirnov test. From the cumulative distribution *F*_*n*_ of the property in the original network, and the joint cumulative distribution *F*_*n*′_ of the randomized networks, a test statistic is derived as *d*_*n*,*n*′_ = sup_*x*_ |*F*_*n*_(*x*) − *F*_*n*′_(*x*)|, where *n* and *n*′ are the number of values in the original, respectively the joint randomized distributions. The *p*-value is 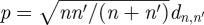.

For each reaction vertex *r*, we determine its centrality, *ν*(*r*), in the original network and in 100 randomized networks, which are obtained by preserving *r* and randomizing the remaining reactions. The *p*-value of *r* is 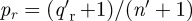, where *q*′_r_ is the number of randomized networks, in which the centrality of *r* is at least as large as in the original network, and *n*′ = 100.

## References

[1] Ciliberti S. Martin O. C. Wagner A. 2007 Innovation and robustness in complex regulatory gene networks. Proc. Natl Acad. Sci. USA 104, 13 591–13 596 10.1073/pnas.0705396104 (doi:10.1073/pnas.0705396104) PMC1959426 17690244

[2] Kuchaiev O. Milenkovic T. Memisevic V. Hayes W. Przulj N. 2010 Topological network alignment uncovers biological function and phylogeny. J. R. Soc. Interface 7, 1341–1354 10.1098/rsif.2010.0063 (doi:10.1098/rsif.2010.0063) 20236959 PMC2894889

[3] Hyduke D. R. Palsson B. Ø 2010 Towards genome-scale signalling-network reconstructions. Nat. Rev. Genet. 11, 297–307 10.1038/nrg2750 (doi:10.1038/nrg2750) 20177425

[4] Duarte N. C. Becker S. A. Jamshidi N. Thiele I. Mo M. L. Vo T. D. Srivas R. Palsson B. Ø 2007 Global reconstruction of the human metabolic network based on genomic and bibliomic data. Proc. Natl Acad. Sci. USA 104, 1777–1782 10.1073/pnas.0610772104 (doi:10.1073/pnas.0610772104) 17267599 PMC1794290

[5] Newman M. E. J. 2003 The structure and function of complex networks. SIAM Rev. 45, 167–256 10.1137/S003614450342480 (doi:10.1137/S003614450342480)

[6] Ravasz E. Somera A. L. Mongru D. A. Oltvai Z. N. Barabási A.-L. 2002 Hierarchical organization of modularity in metabolic networks. Science 297, 1551–1555 10.1126/science.1073374 (doi:10.1126/science.1073374) 12202830

[7] Guimerà R. Amaral L. A. N. 2005 Functional cartography of complex metabolic networks. Nature 433, 895–900 10.1038/nature03288 (doi:10.1038/nature03288) 15729348 PMC2175124

[8] Jeong H. Tombor B. Albert R. Oltvai Z. N. Barabási A.-L. 2000 The large-scale organization of metabolic networks. Nature 407, 651–654 10.1038/35036627 (doi:10.1038/35036627) 11034217

[9] Dorogovtsev S. N. Mendes J. F. F. 2003 Evolution of networks: from biological nets to the Internet and WWW. Oxford, UK: Oxford University Press

[10] Barabási A.-L. Albert R. 1999 Emergence of scaling in random networks. Science 286, 509–512 10.1126/science.286.5439.509 (doi:10.1126/science.286.5439.509) 10521342

[11] Yamada T. Bork P. 2009 Evolution of biomolecular networks: lessons from metabolic and protein interactions. Nat. Rev. Mol. Cell. Biol. 10, 791–803 10.1038/nrm2787 (doi:10.1038/nrm2787) 19851337

[12] Lotka A. J. 1922 Natural selection as a physical principle. Proc. Natl Acad. Sci. USA 8, 151–154 10.1073/pnas.8.6.151 (doi:10.1073/pnas.8.6.151) 16576643 PMC1085053

[13] Caetano-Anolles G. Yafremava L. S. Gee H. Caetano-Anolles D. Kim H. S. Mittenthal J. E. 2009 The origin and evolution of modern metabolism. Int. J. Biochem. Cell Biol. 41, 285–297 10.1016/j.biocel.2008.08.022 (doi:10.1016/j.biocel.2008.08.022) 18790074

[14] Fani R. Fondi M. 2009 Origin and evolution of metabolic pathways. Phys. Life Rev. 6, 23–52 10.1016/j.plrev.2008.12.003 (doi:10.1016/j.plrev.2008.12.003) 20416849

[15] Casella G. Berger R. L. 1990 Statistical inference. Belmont, CA: Duxbury Press

[16] Maslov S. 2007 Complex networks: role model for modules. Nat. Phys. 3, 18–19

[17] Serrano M. A. Boguñá M. Pastor-Satorras R. 2006 Correlations in weighted networks. Phys. Rev. E Stat. Nonlin. Soft Matter. Phys. 74, 055101 10.1103/PhysRevE.74.055101 (doi:10.1103/PhysRevE.74.055101) 17279959

[18] Guimerà R. Sales-Pardo M. Amaral L. A. N. 2007 Classes of complex networks defined by role-to-role connectivity profiles. Nat. Phys. 3, 63–69 10.1038/nphys489 (doi:10.1038/nphys489) 18618010 PMC2447920

[19] Milo R. Shen-Orr S. Itzkovitz S. Kashtan N. Chklovskii D. Alon U. 2002 Network motifs: simple building blocks of complex networks. Science 298, 824–827 10.1126/science.298.5594.824 (doi:10.1126/science.298.5594.824) 12399590

[20] Maslov S. Sneppen K. 2002 Specificity and stability in topology of protein networks. Science 296, 910–913 10.1126/science.1065103 (doi:10.1126/science.1065103) 11988575

[21] Sales-Pardo M. Guimerà R. Moreira A. A. Amaral L. A. N. 2007 Extracting the hierarchical organization of complex systems. Proc. Natl Acad. Sci. USA 104, 15 224–15 229 10.1073/pnas.0703740104 (doi:10.1073/pnas.0703740104) 17881571 PMC2000510

[22] de la Fuente A. Fotia G. Maggio F. Mancosu G. Pieroni E. 2008 Insights into biological information processing: structural and dynamical analysis of a human protein signalling network. J. Phys. A Math. Theoret. 41, 224013 10.1088/1751-8113/41/22/224013 (doi:10.1088/1751-8113/41/22/224013)

[23] Milo R. Itzkovitz S. Kashtan N. Levitt R. Shen-Orr S. Ayzenshtat I. Sheffer M. Alon U. 2004 Superfamilies of evolved and designed networks. Science 303, 1538–1542 10.1126/science.1089167 (doi:10.1126/science.1089167) 15001784

[24] Marr C. Müller-Linow M. Hütt M.-T. 2007 Regularizing capacity of metabolic networks. Phys. Rev. E Stat. Nonlin. Soft Matter. Phys. 75, 041917 10.1103/PhysRevE.75.041917 (doi:10.1103/PhysRevE.75.041917) 17500931

[25] Basler G. Ebenhöh O. Selbig J. Nikoloski Z. 2011 Mass-balanced randomization of metabolic networks. Bioinformatics 27, 1397–1403 10.1093/bioinformatics/btr145 (doi:10.1093/bioinformatics/btr145) 21436128 PMC3087954

[26] Feist A. M. Henry C. S. Reed J. L. Krummenacker M. Joyce A. R. Karp P. D. Broadbelt L. J. Hatzimanikatis V. Palsson B. Ø 2007 A genome-scale metabolic reconstruction for *Escherichia coli* K-12 MG1655 that accounts for 1260 ORFs and thermodynamic information. Mol. Syst. Biol. 3, 121 10.1038/msb4100155 (doi:10.1038/msb4100155) 17593909 PMC1911197

[27] Handorf T. Ebenhöh O. Heinrich R. 2005 Expanding metabolic networks: scopes of compounds, robustness, and evolution. J. Mol. Evol. 61, 498–512 10.1007/s00239-005-0027-1 (doi:10.1007/s00239-005-0027-1) 16155745

[28] Ebenhöh O. Handorf T. 2009 Functional classification of genome-scale metabolic networks. EURASIP J. Bioinform. Syst. Biol. 2009, 1–13 10.1155/2009/570456 (doi:10.1155/2009/570456) PMC3171432 19300528

[29] Borenstein E. Kupiec M. Feldman M. W. Ruppin E. 2008 Large-scale reconstruction and phylogenetic analysis of metabolic environments. Proc. Natl Acad. Sci. USA 105, 14 482–14 487 10.1073/pnas.0806162105 (doi:10.1073/pnas.0806162105) PMC2567166 18787117

[30] Wagner A. Fell D. A. 2001 The small world inside large metabolic networks. Proc. R. Soc. Lond. B 268, 1803–1810 10.1098/rspb.2001.1711 (doi:10.1098/rspb.2001.1711) PMC1088812 11522199

[31] Hubbell C. H. 1965 An input–output approach to clique identification. Sociometry 28, 377–399 10.2307/2785990 (doi:10.2307/2785990)

[32] Langville A. N. Meyer C. D. 2003 Deeper inside PageRank. Internet Math. 1, 335–380 10.1080/15427951.2004.10129091 (doi:10.1080/15427951.2004.10129091)

[33] Lehoucq R. B., Sorensen D. C., Yang C. (1998). Arpack users guide: solution of large scale eigenvalue problems by implicitly restarted Arnoldi methods..

[34] Mesquita A. 2010 Caloric restriction or catalase inactivation extends yeast chronological lifespan by inducing H_2_O_2_ and superoxide dismutase activity. Proc. Natl Acad. Sci. USA 107, 15 123–15 128 10.1073/pnas.1004432107 (doi:10.1073/pnas.1004432107) PMC2930563 20696905

[35] Zamocky M. Furtmüller P. G. Obinger C. 2008 Evolution of catalases from bacteria to humans. Antioxid. Redox Signal. 10, 1527–1548 10.1089/ars.2008.2046 (doi:10.1089/ars.2008.2046) 18498226 PMC2959186

[36] van Loon A. P. Pesold-Hurt B. Schatz G. 1986 A yeast mutant lacking mitochondrial manganese-superoxide dismutase is hypersensitive to oxygen. Proc. Natl Acad. Sci. USA 83, 3820–3824 10.1073/pnas.83.11.3820 (doi:10.1073/pnas.83.11.3820) 3520557 PMC323615

[37] Gralla E. B. Valentine J. S. 1991 Null mutants of *Saccharomyces cerevisiae* Cu, Zn superoxide dismutase: characterization and spontaneous mutation rates. J. Bacteriol. 173, 5918–5920 1885557 10.1128/jb.173.18.5918-5920.1991 PMC208328

[38] Noor R. Mittal S. Iqbal J. 2002 Superoxide dismutase: applications and relevance to human diseases. Med. Sci. Monit. 8, RA210–RA215 12218958

[39] Tashian R. E. 1989 The carbonic anhydrases: widening perspectives on their evolution, expression and function. Bioessays 10, 186–192 10.1002/bies.950100603 (doi:10.1002/bies.950100603) 2500929

[40] Henry R. P. 1996 Multiple roles of carbonic anhydrase in cellular transport and metabolism. Annu. Rev. Physiol. 58, 523–538 10.1146/annurev.ph.58.030196.002515 (doi:10.1146/annurev.ph.58.030196.002515) 8815807

[41] Smith K. S. Jakubzick C. Whittam T. S. Ferry J. G. 1999 Carbonic anhydrase is an ancient enzyme widespread in prokaryotes. Proc. Natl Acad. Sci. USA 96, 15 184–15 189 10.1073/pnas.96.26.15184 (doi:10.1073/pnas.96.26.15184) PMC24794 10611359

[42] Smith K. S. Ferry J. G. 2000 Prokaryotic carbonic anhydrases. FEMS Microbiol. Rev. 24, 335–366 10.1111/j.1574-6976.2000.tb00546.x (doi:10.1111/j.1574-6976.2000.tb00546.x) 10978542

[43] Ferreira F. J. Guo C. Coleman J. R. 2008 Reduction of plastid-localized carbonic anhydrase activity results in reduced arabidopsis seedling survivorship. Plant Physiol. 147, 585–594 10.1104/pp.108.118661 (doi:10.1104/pp.108.118661) 18434607 PMC2409021

[44] Duanmu D. Wang Y. Spalding M. H. 2009 Thylakoid lumen carbonic anhydrase (*CAH3*) mutation suppresses air-dier phenotype of *LCIB* mutant in *Chlamydomonas reinhardtii*. Plant Physiol. 149, 929–937 10.1104/pp.108.132456 (doi:10.1104/pp.108.132456) 19074623 PMC2633820

[45] Gilmour K. M. 2010 Perspectives on carbonic anhydrase. Comp. Biochem. Physiol. A Mol. Integr. Physiol. 157, 193–197 10.1016/j.cbpa.2010.06.161 (doi:10.1016/j.cbpa.2010.06.161) 20541618

[46] Novotny C. P. Englesberg E. 1966 The l-arabinose permease system in *Escherichia coli* B/r. Biochim. Biophys. Acta 117, 217–230 5330661 10.1016/0304-4165(66)90169-3

[47] Schleif R. 2010 AraC protein, regulation of the l-arabinose operon in *Escherichia coli*, and the light switch mechanism of AraC action. FEMS Microbiol. Rev. 34, 779–796 20491933 10.1111/j.1574-6976.2010.00226.x

[48] Irani M. Maitra P. K. 1974 Isolation and characterization of *Escherichia coli* mutants defective in enzymes of glycolysis. Biochem. Biophys. Res. Commun. 56, 127–133 10.1016/S0006-291X(74)80324-4 (doi:10.1016/S0006-291X(74)80324-4) 4595969

[49] Oh Y. K. Freese E. 1976 Manganese requirement of phosphoglycerate phosphomutase and its consequences for growth and sporulation of *Bacillus subtilis*. J. Bacteriol. 127, 739–746 182667 10.1128/jb.127.2.739-746.1976 PMC232979

[50] Lam K. B. Marmur J. 1977 Isolation and characterization of *Saccharomyces cerevisiae* glycolytic pathway mutants. J. Bacteriol. 130, 746–749 400791 10.1128/jb.130.2.746-749.1977 PMC235276

[51] Papini M. Nookaew I. Scalcinati G. Siewers V. Nielsen J. 2010 Phosphoglycerate mutase knock-out mutant *Saccharomyces cerevisiae*: physiological investigation and transcriptome analysis. Biotechnol. J. 5, 1016–1027 10.1002/biot.201000199 (doi:10.1002/biot.201000199) 20815084

[52] Feist A. M. Zielinski D. C. Orth J. D. Schellenberger J. Herrgård M. J. Palsson B. 2010 Model-driven evaluation of the production potential for growth-coupled products of *Escherichia coli*. Metab. Eng. 12, 173–186 19840862 10.1016/j.ymben.2009.10.003 PMC3125152

[53] Oh Y.-K. Palsson B. Park S. M. Schilling C. H. Mahadevan R. 2007 Genome-scale reconstruction of metabolic network in *Bacillus subtilis* based on high-throughput phenotyping and gene essentiality data. J. Biol. Chem. 282, 28 791–28 799 10.1074/jbc.M703759200 (doi:10.1074/jbc.M703759200) 17573341

[54] Herrgård M. J. 2008 A consensus yeast metabolic network reconstruction obtained from a community approach to systems biology. Nat. Biotechnol. 26, 1155–1160 10.1038/nbt1492 (doi:10.1038/nbt1492) 18846089 PMC4018421

[55] May P. 2008 Metabolomics- and proteomics-assisted genome annotation and analysis of the draft metabolic network of *Chlamydomonas reinhardtii*. Genetics 179, 157–166 10.1534/genetics.108.088336 (doi:10.1534/genetics.108.088336) 18493048 PMC2390595

[56] Rhee S. Y. 2003 The arabidopsis information resource (TAIR): a model organism database providing a centralized, curated gateway to arabidopsis biology, research materials and community. Nucleic Acids Res. 31, 224–228 10.1093/nar/gkg076 (doi:10.1093/nar/gkg076) 12519987 PMC165523

[57] Ma H. Sorokin A. Mazein A. Selkov A. Selkov E. Demin O. Goryanin I. 2007 The Edinburgh human metabolic network reconstruction and its functional analysis. Mol. Syst. Biol. 3, 135 10.1038/msb4100177 (doi:10.1038/msb4100177) 17882155 PMC2013923

[58] Dobson C. M. 2004 Chemical space and biology. Nature 432, 824–828 10.1038/nature03192 (doi:10.1038/nature03192) 15602547

[59] Horowitz E. Sahni S. 1974 Computing partitions with applications to the knapsack problem. J. ACM 21, 277–292 10.1145/321812.321823 (doi:10.1145/321812.321823)

